# Selection of optimal recording sites for limited lead body surface potential mapping: A sequential selection based approach

**DOI:** 10.1186/1472-6947-6-9

**Published:** 2006-02-17

**Authors:** Dewar D Finlay, Chris D Nugent, Mark P Donnelly, Robert L Lux, Paul J McCullagh, Norman D Black

**Affiliations:** 1 School of Computing and Mathematics, Faculty of Engineering, University of Ulster, Belfast, Northern Ireland; 2 Nora Eccles Harrison Cardiovascular Research and Training Institute, University of Utah, Slat Lake City, USA

## Abstract

**Background:**

In this study we propose the development of a new algorithm for selecting optimal recording sites for limited lead body surface potential mapping. The proposed algorithm differs from previously reported methods in that it is based upon a simple and intuitive data driven technique that does not make any presumptions about deterministic characteristics of the data. It uses a forward selection based search technique to find the best combination of electrocardiographic leads.

**Methods:**

The study was conducted using a dataset consisting of body surface potential maps (BSPM) recorded from 116 subjects which included 59 normals and 57 subjects exhibiting evidence of old Myocardial Infarction (MI). The performance of the algorithm was evaluated using spatial RMS voltage error and correlation coefficient to compare original and reconstructed map frames.

**Results:**

In all, three configurations of the algorithm were evaluated and it was concluded that there was little difference in the performance of the various configurations. In addition to observing the performance of the selection algorithm, several lead subsets of 32 electrodes as chosen by the various configurations of the algorithm were evaluated. The rationale for choosing this number of recording sites was to allow comparison with a previous study that used a different algorithm, where 32 leads were deemed to provide an acceptable level of reconstruction performance.

**Conclusion:**

It was observed that although the lead configurations suggested in this study were not identical to that suggested in the previous work, the systems did bear similar characteristics in that recording sites were chosen with greatest density in the precordial region.

## Background

Body Surface Potential Mapping refers to the process of acquisition and display of temporal and spatial distributions of electrocardiographic potentials recorded from multiple sites on the torso [1]. In contrast to more conventional recording techniques, such as the 12 lead ECG, where scalar traces allow for assessment of wave amplitudes, intervals and morphology, Body surface potential maps (BSPM) are assessed based on the shape of their contours, number and location of extrema and the dynamics of each [2]. As well as providing the ability to locate electrocardiographic events in both space and time, BSPMs yield more diagnostic content by capturing information at anatomical regions not interrogated by the widely utilised 12 lead ECG. This allows for more comprehensive diagnosis of conditions such as Myocardial Infarction (MI), Wolff-Parkinson-White syndrome and Ventricular tachycardia [3-5].

As the objective is to accurately display total body surface potential distributions, systems that use up to and in excess of 200 recording sites, covering the entire thoracic surface, have been proposed [6]. Such an abundance of sampled channels means that virtually all Electrocardiogram (ECG) information as projected onto the body surface is captured. However, although desirable the practical limitations of such an approach are immediately obvious as the need to apply approximately 200 surface electrodes to a subject is enough alone to make the technique highly infeasible in routine clinical practice. For this reason, and based on the appreciation that there is redundancy in BSPMs [7] there is an interest in trying to find the optimal number and position of recording sites required to capture the necessary information required to allow visualisation and interpretation of total body surface potential distributions.

In the current study we propose a new method for finding the optimal location of recoding sites in 192 lead BSPMs. The proposed algorithm differs from the existing techniques in that it is based upon a simple and intuitive data driven technique that does not make any presumptions about deterministic characteristics of the data.

The most prolific works in this area have been that of Barr [8] and Lux [9]. Both of these investigators have proposed strategies to locate of the minimum number of recording sites required to capture the maximum amount of ECG information. In the work of Barr [8] 150 lead BSPMs were analysed and a technique based upon principal component analysis was proposed. This study concluded that only 24 of the 150 recording sites were necessary to capture the relevant ECG information. In the subsequent study by Lux [9] a more intuitive method of analysing correlation and energy content in the given signals was adopted and through analysis of 192 leads BSPMs this study found that a subset of between 30–35 optimally positioned recording sites was sufficient. In both studies, the accuracy of selected recording sites were evaluated by testing how well the signals at the chosen sites could be used to estimate the signals at the sites that were not chosen.

Before describing the proposed algorithm one must appreciate the notion of a statistical transformation of electrocardiographic leads. This concept is based upon the work described in [10] where the notion of relating one lead system to another by means of a set of statistically derived coefficients was introduced. Based on the assumption that all the independent information is captured by the recorded leads it was shown that a linear combination of the recorded signals can be used to estimate signals at sites which have not been recorded. Although [10] focused specifically on vectorcardiography, this principle can be extended to other recording systems and a prominent example of this is the commercially available EASI lead system, originally proposed by Dower [11]. This system uses 3 bipolar measurements, recorded from just 4 recording sites to derive the 12-lead ECG. In general and regardless of the ECG lead system considered, the relationship between a number of recorded sites and some estimated site can be described as:

*ecg_e _*= *α *• *ecg*_1 _+ *β *• *ecg*_2 _+ *λ *• *ecg*_3 _+ ........     (1)

Where *ecg*_*e *_is the estimated ECG signal, *ecg_1_*, *ecg_2 _*and *ecg_3_*, are the measured signals, and *α*, *β *and *λ *are a set of coefficients that weight the measured signals. It should be noted that the coefficients, *α*, *β *and *λ*, remain constant over time and each set of these coefficients is unique to the site being estimated. In the current study transformation coefficients are determined using multiple linear regression (MLR) by least mean squares.

## Methods

### Sequential selection algorithm

The algorithm proposed in this study builds a limited lead set by iteratively evaluating and selecting the available sites based on their combined ability to estimate entire surface potential distributions. The process begins by evaluating how well each of the 192 available recording sites can be used individually to reconstruct a set of BSPMs. Although the notion of estimating entire surface potential distributions from just one recording site may seem nonsensical, this is merely the first step in our algorithm and it is the combination of this 'best' site and further selected sites that are significant. The process is then repeated and on the second pass each of the remaining 191 sites are evaluated in conjunction with the previously selected site. The site that works best in conjunction with the first chosen site is then added to the limited lead set and the process is repeated again. At this point we have found the best two sites for reconstructing BSPMs and we are looking for the third best site. On this third iteration the remaining 190 sites are evaluated in conjunction with the lead sub set which now consists of two sites. This process is repeated until some stopping criteria has been met, e.g. an acceptable level of accuracy has been attained, or the desired number of recording sites has been reached.

### Map evaluation

On each iteration the algorithm must evaluate how well the given site(s) can reconstruct a set of BSPMs. Although the method of determining the transformation has been established (MLR) it is still necessary to provide some measure of how well the reconstructed map frames compare with the actual recorded map frames. This measure of similarity is used to rank the available recording sites for selection and as this was primarily a study in limited lead mapping the objective was to make comparison based on the accuracy of spatial distributions. To compare the spatial distributions two different measures, spatial RMS voltage error and Correlation Coefficient have previously been reported [7,9,12].

#### Spatial RMS voltage error

This measure is used to provide the average potential error at each of the estimated sites not included in the limited lead array. If *P*_1 _and *P*_2 _are the vectors of the measured and estimated potentials respectively, and *n *is the number of sites at which potentials have been estimated, the spatial RMS error *e *can be determined by the equation

e=|P1−P2|n     (2)
 MathType@MTEF@5@5@+=feaafiart1ev1aaatCvAUfKttLearuWrP9MDH5MBPbIqV92AaeXatLxBI9gBaebbnrfifHhDYfgasaacH8akY=wiFfYdH8Gipec8Eeeu0xXdbba9frFj0=OqFfea0dXdd9vqai=hGuQ8kuc9pgc9s8qqaq=dirpe0xb9q8qiLsFr0=vr0=vr0dc8meaabaqaciaacaGaaeqabaqabeGadaaakeaacqWGLbqzcqGH9aqpdaWcaaqaamaaemaabaGaemiuaa1aaSbaaSqaaiabigdaXaqabaGccqGHsislcqWGqbaudaWgaaWcbaGaeGOmaidabeaaaOGaay5bSlaawIa7aaqaamaakaaabaGaemOBa4galeqaaaaakiaaxMaacaWLjaWaaeWaaeaacqaIYaGmaiaawIcacaGLPaaaaaa@3D0D@

In previous studies this figure has been directly compared to the estimated system noise providing an indication of the minimum number of leads required to accurately reconstruct total surface distributions [9,12].

#### Correlation coefficient

This provides a measure of the similarity in map patterns between measured and estimated map frames independent of amplitude. If *P *is the original map frame and *P' *is the estimated map frame the Correlation Coefficient *ρ *can be described as

ρ=P.P′|P||P′|     (3)
 MathType@MTEF@5@5@+=feaafiart1ev1aaatCvAUfKttLearuWrP9MDH5MBPbIqV92AaeXatLxBI9gBaebbnrfifHhDYfgasaacH8akY=wiFfYdH8Gipec8Eeeu0xXdbba9frFj0=OqFfea0dXdd9vqai=hGuQ8kuc9pgc9s8qqaq=dirpe0xb9q8qiLsFr0=vr0=vr0dc8meaabaqaciaacaGaaeqabaqabeGadaaakeaacqaHbpGCcqGH9aqpdaWcaaqaaiabdcfaqjabc6caUiqbdcfaqzaafaaabaWaaqWaaeaacqWGqbauaiaawEa7caGLiWoadaabdaqaaiqbdcfaqzaafaaacaGLhWUaayjcSdaaaiaaxMaacaWLjaWaaeWaaeaacqaIZaWmaiaawIcacaGLPaaaaaa@3F27@

This measure is useful as small errors of amplitude on low amplitude map frames may appear to be insignificant, yet pattern differences might be extreme.

### Algorithm configuration

The fact that two measures of accuracy exist mean that there are potentially two ways in which the recording site selection process could be guided. If the spatial RMS error is used in the choice of each recording sites on each pass of the algorithm it is possible in theory that recording sites would be chosen to favour minimal potential error. On the other hand if the correlation coefficient is used it is possible that the lead subset shall be chosen to favour similarities in map patterns. In the initial experiments the spatial RMS error was chosen to guide the selection, this is based on the assumption that as this error approaches zero map frame patterns will increase in similarity until they are identical, in contrast when the correlation coefficient is equal to 1 (identical patterns) this has in theory no bearing on amplitude differences.

The remaining consideration in the development of the algorithm was the choice of the minimum number of recording sites that would provide an acceptable level of reconstruction performance. As this study is primarily focused on the development and evaluation of a lead selection algorithm as opposed to the evaluation of some proposed lead set, we based our stopping criteria on results already presented in the literature. As the studies by Barr [8] and Lux [9] suggest somewhere in the region of between 24–35 recording sites, we elected that our algorithm would terminate after 40 iterations, this being adequately in excess of the numbers proposed in the previous studies. Although the algorithm was configured to terminate after 40 iterations we were keen to provide some comparison of the suggested lead subsets with the 32 lead subsets proposed in [12]. This is of particular interest as the data used in that particular study is of the same format as the data used here, allowing direct comparison of results. For that reason, all suggested lead subsets in this study shall consist of 32 recording sites.

### Clinical data

**Figure 1:**
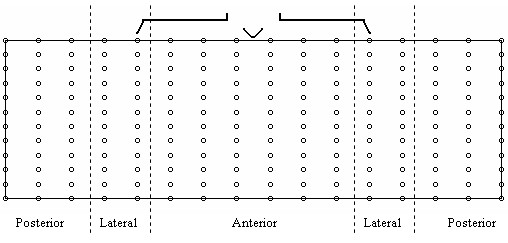
**192 Electrode Array**. Schematic representation of the 192 electrode array, depicted as an unrolled cylindrical matrix. The middle region correspond with the anterior torso and the left and right regions correspond with the posterior.

In the current study a dataset consisting of 192 lead BSPMs recorded from 116 subjects was used. This was made up of a group of 59 normal subjects, exhibiting no disease symptoms and a group of 57 subjects exhibiting electrocardiographic evidence of old MI at various locations, see Table 1. The recording procedure involved placing 192 electrodes, in 16 equi-spaced columns of 12 electrodes, around the thoracic circumference of each subject. All 192 channels of information were recorded simultaneously at 1000 Hz for several seconds (5–10 cardiac cycles). Subsequent to recording; the information from each channel was reduced to represent one beat. This was achieved by taking the average of the cardiac cycles recorded on each channel. A schematic of the electrode array is illustrated in Figure 1. These data were acquired during the course of NIH funded research, at the University of Utah, Salt Lake City, under the direction of Professor Robert Lux. All data acquisition was managed by an ECG technician under the supervision of a board certified cardiologist who was responsible for recruiting patients and subjects for the study. The University of Utah Institutional Review Board approved these studies. The process of acquiring the data used in this study is also described in [7,9,12].

**Table 1:** Data set breakdown detailing infarct locations.

Normals		**59**
Myocardial Infarction		**57**
Inferior	30	
Anterior	14	
Posterior	2	
Aterolateral	8	
Inferolateral	2	
Inferior-posterior	1	
Total		**116**

All lead selection experiments were conducted on a randomly selected subset of recordings from 87 of the 116 subjects available. From each subject's recording all QRS frames were used and every fifth STT frame was used resulting in a total of 13157 individual map frames. This down sampling of STT information reduces computational load and is viable due to the fact that STT potential distributions are usually simpler than QRS distributions [9]. On each selection run 3 quarters of this data (3 out of every 4 frames) were used to build the transformation for each recording site under evaluation with the remaining quarter (1 in every 4) used to evaluate the transformation. Although this evaluation of the transformation is independent, the measures of accuracy obtained cannot be used to suggest the final accuracy of the lead system as these measures will have been used to guide the selection process. In order to eliminate the possibility of such bias the data from the remaining 29 subjects was used to evaluate the lead subset. Again the same method of sampling as applied to the training set was applied to the test set resulting in a total of 4381 map frames.

## Results

**Figure 2:**
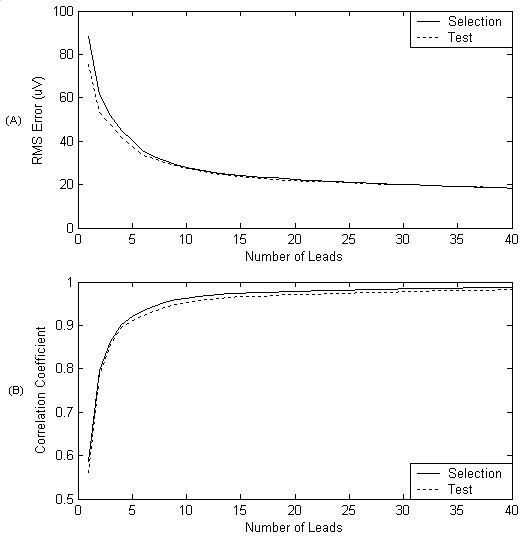
**Performance of algorithm when spatial RMS error is used to guide the selection**. (a) depicts spatial RMS error during the selection process (train) versus spatial RMS error on unseen test data. (b) Depicts correlation coefficient during selection (train) versus test.

**Figure 3:**
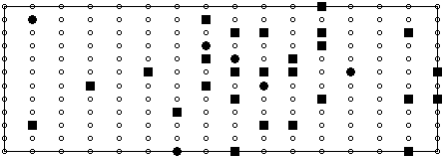
**Positions of the top 32 recording sites**. Here the positions of the top 32 recording sites are shown. The filled circles indicate the first 6 chosen sites and the remaining 26 sites are indicated using filled squares.

**Figure 4:**
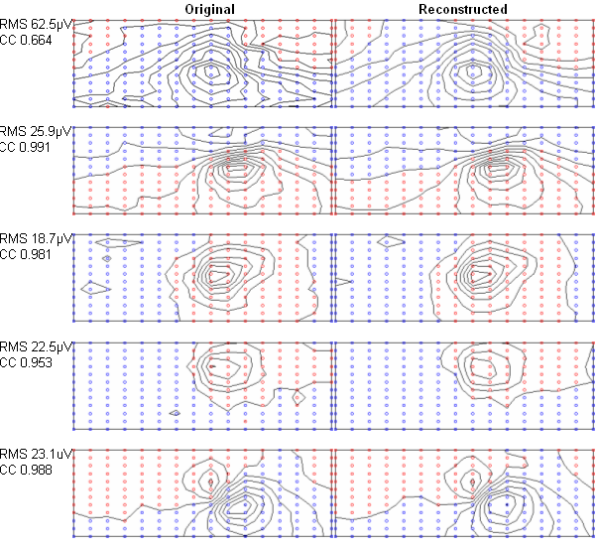
**Sample of original and reconstructed map frames**. Here a selection of original and reconstructed map frames are shown. The estimated frames have been reconstructed from the 32 recording sites depicted in Figure 3. RMS and CC denote values of RMS voltage error and correlation coefficient respectively.

The results of the initial lead selection experiment are illustrated in Figure 2. Here the performance of the selection process and the performance of the selected leads on unseen data are depicted. It should be noted that although both RMS error and correlation coefficient are plotted for the selection process, only the RMS error was used in guiding the selection. In Figure 3 we have indicated the positions of the first 32 recording sites. Additionally, to allow comparison of the positions of recording sites in more conventional ECG approaches the positions of the first 6 sites that the algorithm chose have been indicated using a different marker. In order to demonstrate the ability of these 32 recording sites in reconstructing a variety of map frames, Figure 4 shows a selection of original map frames in comparison to those that have been estimated from the 32 recording sites.

**Figure 5:**
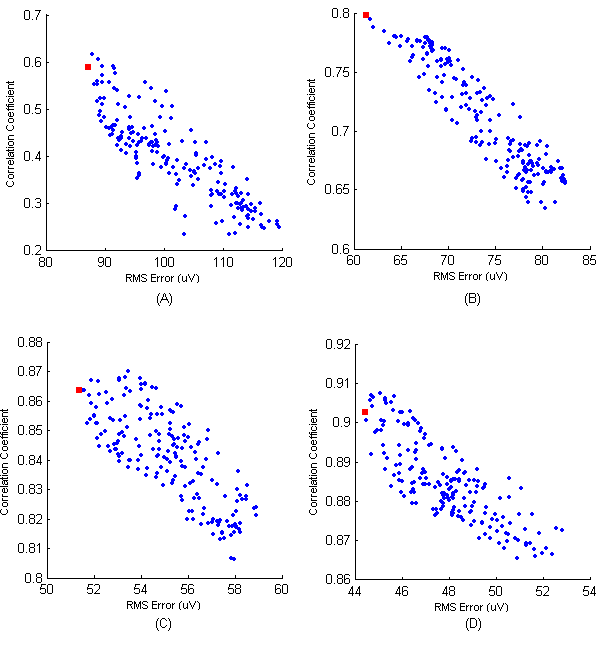
**Indication of lead dominance when both spatial RMS error and correlation coefficient are considered**. Scatter plot of RMS error versus correlation coefficient for first four passes of selection algorithm. These plots indicate that on each pass the best place lead in terms of RMS error is not the best placed lead in terms of correlation coefficient.

In order to asses the effect of using the spatial RMS error to guide the lead selection, we set about analysing the data that was generated during the previous selection run. To establish if there was a difference between the best sites in terms of RMS error and the best sites in terms of correlation coefficient a series of scatter plots were constructed. These scatter plots depict the RMS error versus correlation coefficient on each pass of the algorithm. The first four of these have been plotted in Figure 5. The first scatter plot, Figure 5a, consists of 192 points and depicts the performance of each recording site on the first pass of the algorithm. The second, Figure 5b, depicts the performance of each recording site on the second iteration and as one site has already been chosen at this stage there are now 191 points. The increase in performance in this second scatter is also evident from the change in scale. The third and forth scatters, Figures 5c and 5d, consist of 190 and 189 points respectively. The key observation from these scatters is that there is a significantly proportional decrease in RMS error as correlation coefficient increases; however it is also obvious from three out of the four examples that the best lead on each pass in terms of RMS error is not the best lead in terms of correlation coefficient. Taking Figure 5a as an example it can be seen that the best recording site in terms of RMS error, highlighted as a red square, is in fact the 3^rd ^best resting site in terms of correlation coefficient. On the analysis of further scatter plots it was found that there were very few passes/iterations where there was one dominant solution, i.e. best in terms of both correlation coefficient and RMS error. Although it is apparent that choosing the best recording site for one criterion results in a reasonably good performance in the other criteria, there was the concern that veering towards one criteria on each pass of the algorithm may lead to a final solution that is significantly biased toward the chosen criteria.

**Figure 6:**
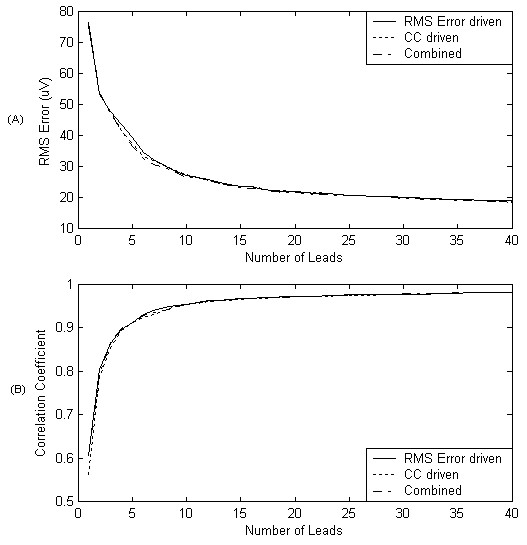
**Comparison of algorithm configurations**. Comparison of selection algorithms performance when the three evaluation methods (RMS error, correlation coefficient, multi-objective) are used to guide the search.

**Figure 7:**
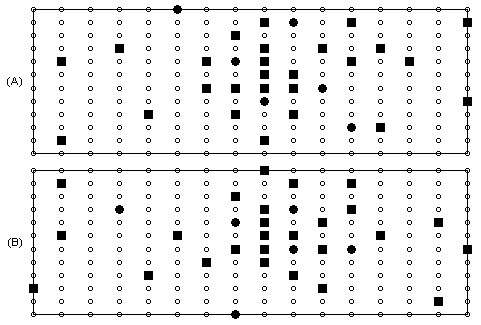
**Top 32 recording sites using various algorithm configuration**. Recording sites chosen using (a) correlation coefficient based evaluation and (b) multi-objective evaluation. In each case the best 32 recording sites are highlighted, with the first six shown using closed circles.

In order to fully appreciate the effects of this observation a further two experiments were conducted. In the first the selection algorithm was reconfigured to use the correlation coefficient on each iteration, and in the second a multi-objective combination approach was introduced. Here, on each iteration, as each recording site was evaluated it was given a rank based on its RMS error and its correlation coefficient. The two ranks for each site were then added to produce a further score and the site with the lowest score was chosen as the best. The results from these further experiments in addition to the result from the first RMS based experiment are illustrated in Figure 6. Although it is difficult to make a statistical comparison between these curves due to the accumulative nature of the underlying data, it can be seen that, on visual inspection, the curves are similar to the extent where it is difficult to distinguish between them. In Figure 7 the locations of the top 32 leads for the further two experiments are shown.

In addition to comparing the performance of the three configurations of the algorithm the performance of the 32 lead subset proposed in [12] was tested using our dataset. Although performance figures were provided in the study in [12] these were obtained using a different data set, and a different method for determining the transformation coefficients making comparison difficult. The results of our comparison are listed in Table 2. In this table the presented values indicate the median of correlation coefficient and RMS error across all reconstructed map frames.

**Table 2:** Comparison of performance of 32 lead subsets. The performance of the three subsets proposed in this study are summarised along with the performance of the Lux 32 lead system [12]. In each case the results are presented as median and interquartile range.

	CC	RMS Error (*μ*V)
RMS	0.978 (0.933–0.992)	19.7 (14.7–31.5)
CC	0.978 (0.933–0.992)	19.7 (14.5–30.6)
MO	0.980 (0.936–0.992)	19.4 (14.4–30.1)
Lux	0.980 (0.935–0.993)	19.4 (14.4–30.2)

## Discussion

The first point of discussion relates to the visual comparison of the original and estimated map frames as shown in Figure 4. These map frames have been estimated from the 32 lead subset chosen with the algorithm guided using RMS error. In assessing the similarity of these example maps it can be seen that in all cases the locations of maxima and minima are almost identical. This is an important criterion as the locations and dynamics of these extrema over time provide the basis for diagnostic discrimination. The discrepancy between the original and estimated map frames is more apparent in the shape of the contours and an interesting observation is that, on a whole, the estimated map frames tend to have smoother contour lines. It assumed that the more jagged contours of the original measured maps are a result of recording artefact and noise, and it would appear that the transformation coefficients, although representing the overall map patterns well, exhibit a filtering effect by not representing this noise. When the reconstruction performance across all map frames in the population was observed, it was evident that on high amplitude frames (particularly QRS frames) the RMS voltage error was generally higher than that obtained on low amplitude map frames. This was in contrast to the correlation coefficient which actually improved on high amplitude frames, an observation similar to that noted by previous investigators [12]. Although examples of reconstructed maps from the selection experiments that used the correlation coefficient and the multi-objective approach have not been included, the resulting maps from these experiments bore similar characteristics to the maps illustrated here.

**Figure 8:**
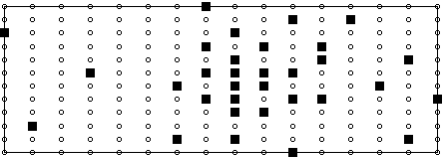
Top 32 recording sites as chosen by Lux.

Although this study is focused more upon the development of a lead selection algorithm as opposed to suggesting actual lead sets for clinical utilisation, the lead set suggested by each configuration of the selection algorithm could be considered as an optimal lead set for the studied population. Observing the suggested lead configurations, as illustrate in Figure 3 and Figure 7, it can be seen that although there is little discrepancy in performance across the three selection methods there are no two electrode configurations that are identical. Across the three sets of 32 sites selected, there were just 7 sites that are common to all three methods, 5 of which also appear in the 32 sites chosen by Lux, illustrated in Figure 8. In comparison of each of the methods with the sites chosen by Lux, the RMS driven method suggested 12 sites in the same locations and the correlation coefficient driven and multi-objective methods each had 11 sites in the same positions as that of Lux. Despite these discrepancies, the general characteristics of each subset are the same with more electrodes being suggested on the anterior surface than on the posterior in all three examples. In addition to this, in each configuration the highest concentration appears to be around the precordial area close to the heart. Considering the positions of the first six recording sites, it can be seen that on each of the suggested lead sets there are at least 4 electrodes positioned anteriorly and in the case of the lead set chosen using the correlation coefficient method (Figure 7a) 5 sites are positioned on the anterior surface. In each case there are at least two sites that are not positioned in close proximity to the locations of the 6 unipolar/precordial electrodes in the conventional 12 lead ECG. Regardless of the exact positions of the recording sites, it is evident from the results presented in Table 2 that there is a similar level of performance across all suggested sub sets of 32 recording sites.

Although the selection algorithm appears to yield convincing results, a valid concern may be expressed regarding the computational cost of such an intense search method. This relates to the fact that as each recording site subset is being evaluated a set of transformation coefficients must be generated to allow the reconstruction of the original BSPM frames from that subset. As stated, in this study this transformation was determined through the method of MLR using least mean squares. To appreciate the computational intensity one must consider that as each subset of recording sites is being evaluated an individual set of coefficients (MLR model) is required to relate the subset to each site to be estimated. Taking the first pass of the algorithm as an example, evaluation of each individual site requires the development of 191 MLR models which are needed to predict the potentials at the respective 191 remaining sites. The first pass through the 192 available recording sites therefore requires the development of 191 × 192 = 36672 unique coefficients. Although this seems a large number the fact that each of the first MLR models consists of only one independent variable means that the calculations required are not intense. The computational load does increase however when the number of sites that have been selected increases, as although there are fewer models to be developed (fewer sites to be estimated) the models are much larger. If a practical lead system were being developed it is highly unlikely that the number of recording sites would ever exceed a few dozen. Indeed it is possible that an algorithm such as this could be used in non-mapping systems where the location of just a few recording sites might be necessary.

This latter point leads us to the next point of discussion which is the possibility of using this algorithm in non mapping applications. One merit of the proposed algorithm is its sequential characteristics which provide greater control over how the algorithm progresses. A practical example of where this advantage could be capitalised upon would be if it was necessary to enforce the positions of several recording sites before allowing the algorithm to select further sites. A situation like this may arise if an existing electrode system was being redeveloped and the designer wished to maintain some of the locations used in the original system whilst selecting new positions that allow maximum information capture. A further scenario may be where it is necessary to exclude certain areas of the torso from the selection process. For example, a practical lead system may be considered as one that did not include posterior recording sites. If such a configuration were desired the proposed algorithm could be configured to only consider the performance of anterior sites neglecting any on the posterior.

Although this algorithm is somewhat different from that previously reported in this domain, there are techniques that have been used in other applications that can be compared. Firstly, similarities can be drawn between this approach and the wrapper approach to feature selection [13], where the best features, or variables, are chosen for classification based on how well they work accumulatively with a given classifier. In the wrapper approach consideration is given to what classifier is used to evaluate the performance of a given feature subset and to what search strategy is used to add or remove features from the feature subset. An analogy can be drawn between the classifier in the wrapper approach and the transformation method used in this approach, and a similar analogy exists between the search strategy in the wrapper approach and the iterative sequential selection method used here. In fact, the iterative sequential selection method used here is almost identical to the Sequential Forward Selection (SFS) method common in the wrapper approach where features are added one by one based on their accumulative classification performance. An equally common approach in the wrapper context is that of Sequential Backward Elimination (SBE) where the process starts with all features present and eliminates them one by one again based on the performance of what remains in the feature subset. The SBE is not a suitable option in this application as the requirement in the initial iterations to build MLR models with all recording sites included would be prohibitive in terms of computational intensity. The algorithm proposed in this study also bears similarities to Stepwise Multiple Regression (SMR) where the contributions of independent variables to one dependant variable are considered and the independent variables that do not contribute significantly are eliminated. There is, however, a subtle difference as this application requires the evaluation of the contribution of independent variables to a number of dependent variables (recording sites) considered together as total surface distributions. Therefore if SMR was applied in this application a different set of recording sites could potentially be suggested for estimating each of the remaining sites.

## Conclusion

In this study we have proposed an algorithm that allows recording sites to be selected based on their actual performance when estimating total body surface potential distributions. From the outset this algorithm was intended to be intuitive and it relied only on the presented data. It has been demonstrated that the performance of the resulting electrode configurations developed by the algorithm perform comparably with lead sets that have been suggested using similar data but using a different technique.

In the assessment of the various configurations of the algorithm, in terms of the metric used to guide the selection of the recording sites, it was observed that although the locations of the resulting sites did vary, there was little or no difference in the reconstruction performance of any of the subsets. For this reason we conclude that either RMS voltage error, correlation coefficient or the multi-objective approach can be used to guide the algorithm.

Considering the lead configurations that have been proposed, although these recording sites have been chosen using a dataset that only represents normal subjects and those who have had MI, it is valid to suggest that any of the three configurations that were proposed would be adequate for use in recording data from subjects from these diagnostic backgrounds.

As a final point it should be stressed that in this study we have proposed an algorithm that picks recording sites based on their ability to accurately estimate BSPMs, and although we can assume that these sites are those which exhibit the most independent information, it has been suggested that there is a distinction between this 'signal' information and diagnostic information [14]. For this reason we can only assume that our proposed lead systems are only optimal for capturing signal information as diagnostic information has not been considered.

## Competing interests

The author(s) declare that they have no competing interests.

## Authors' contributions

DDF was responsible for designing the study, conducting all experimental procedures, and along with CDN, MPD and RLL, interpreting and presenting the results. Both DDF and CDN participated equally in drafting the manuscript. PJM and NDB participated in the design of the study and also helped to draft the manuscript.

## Pre-publication history

The pre-publication history for this paper can be accessed here:


